# When twice is better than once: increased liking of repeated items influences memory in younger and older adults

**DOI:** 10.1186/s40359-021-00531-8

**Published:** 2021-02-06

**Authors:** Rocco Palumbo, Alberto Di Domenico, Beth Fairfield, Nicola Mammarella

**Affiliations:** 1 Department of Psychological, Health and Territorial Sciences (DiSPuTer), G. d’Annunzio University of Chieti-Pescara, Via dei Vestini 31, 66100 Chieti, Italy; 2 Department of Neurology, Boston University, School of Medicine, Boston, MA USA

**Keywords:** Emotional memory, Positivity effect, Repetition

## Abstract

**Background:**

Numerous studies have reported that the repeated presentation of a stimulus leads to an increase in positive affect towards the stimulus itself (the so-called *mere exposure effect*). Here, we evaluate whether changes in liking due to repetition may have a differential impact on subsequent memories in younger and older adults.

**Method:**

In two experiments, younger and older adults were asked to rate a series of nonwords (Experiment 1) or unfamiliar neutral faces (Experiment 2) in terms of how much they like them and then presented with a surprise yes–no recognition memory task. At study, items were repeated either consecutively (massed presentation) or with a lag of 6 intervening items (spaced presentation).

**Results:**

In both experiments, participants rated spaced repeated items more positively than massed items, i.e. they liked them most. Moreover, older adults remembered spaced stimuli that they liked most better than younger adults.

**Conclusions:**

The findings are discussed in accordance with the mechanisms underlying positivity effects in memory and the effect of repetition on memory encoding.

## Background

Decades of studies have shown that repeated exposure to a stimulus can lead individuals to consider the stimulus as more pleasant [1]. This effect, known as the *mere exposure effect,* suggests that information deriving from repetitions may have an impact on the cognition-emotion interaction. Repetition, in fact, may carry a positive connotation and/or orientate emotional reactions towards the positive pole. In this study, we assume that repetition influences emotional preferences (like/dislike ratings) and that these preferences differentially affect subsequent memory. The fundamental question is whether repetition itself may generate a positivity bias (measured here in terms of an increase in liking) that may resemble the “age by valence” interaction typically shown in emotional memory studies (e.g., the so-called *positivity effect*) [2].

To this end, we conducted two experiments varying the unfamiliar material to be studied. Nonwords were used in experiment 1, whereas unfamiliar faces were used in Experiment 2. In both experiments, the main manipulation was repetition since stimuli were presented once or twice. After rating the liking of a list of items, younger and older adults were asked to remember whether each item was old or new.

### Memory, repetition and the mere exposure effect

Decades of experimental studies have shown that repetition leads to better memory [3]. This effect can be explained from a cognitive perspective where repetition strengthens memory traces that subsequently aid individuals during retrieval [4]. Nonetheless, not all types of repetition have been shown to be effective. Indeed, items are remembered better when they are repeated after one or more intervening items from the first occurrence (*spaced* presentation) compared to an item that immediately follows its first occurrence (*massed* presentation). This so-called *spacing effect* is a very robust phenomenon and has been observed in many memory tasks including free recall, recognition, cued recall, and frequency estimation [5, 6]. The explanation for the spacing effect is certainly multifactorial but the main account posits repetition priming mechanisms that operate in between the two presentations of a target. In particular, the first presentation of a target primes the second occurrence of the target item, thus reducing its semantic processing. Moreover, these repetition priming effects are stronger when the delay between the prime and the target is short [7], leading to more impairment in massed presentations than in spaced presentations as spaced items undergo more extensive semantic processing than massed items. In turn, this provides the basis for the emergence of the spacing effect.

Repetition has also been shown to influence affective processing [8]. In fact, the mere exposure effect also occurs when the liking for a stimulus increases following repeated exposure to that stimulus. Among the many accounts advanced to explain the effect [1], the processing fluency theory fits well with our hypothesis, that is, spacing effects based on repetition priming mechanisms. According to processing fluency, the second time we experience a stimulus, it is processed more easily than novel stimuli. Consequently, repetition increases the ease and speed of processing of presented stimuli. Such fluency then causes an increase in liking because fluency produces a sense of familiarity and what is familiar is generally perceived as more positive [9]. There are multiple variations of the processing fluency approach, but what is relevant here is that the level of fluency may differ across stimuli and this may impact emotional memories of younger and older adults in different ways. We describe our predictions better in the section below.

### Aging, repetition, emotion-memory interaction

Studies on the mere exposure effects in aging are few. Wiggs [10], for instance, conducted one of the first experiments that investigated the mere exposure effect in a group of younger and older adults and found that both groups were sensitive to the frequency of occurrence (Exp. 3). Another study by Winograd and colleagues [11] found the mere exposure effect in patients with Alzheimer's disease suggesting spared implicit processing effects in pathological aging. In general, theoretical models of the mere exposure effects suggest that repetition acts as an automatic prompt for the positive evaluation of a stimulus in both younger and older adults. Generally speaking, these data are consistent with prior work on familiarity and aging [12, 13] as older adults have been shown to like or endorse fame to familiar items. In fact, older adults were more likely to remember old non-famous names as famous when faces were represented. Thus, the repetition of a stimulus may increase familiarity and, consequently, liking for the stimulus. After a retention interval, when the stimulus is presented again, liking for the stimulus increases. Our assumption is that this preference may impact later memory for the stimulus itself. Here, we aimed to investigate whether repetition modulates memory in the following ways.

First, we adopted a spacing effect manipulation to simulate mere exposure effects in a single study phase. Items were repeated either consecutively or with a number of intervening items. The rationale being as follows. The mere exposure effect has been considered an example of repetition priming effects [1, 14–17] and it is based on the general assumption that processing is facilitated when a stimulus has been previously encountered. Following Butler et al.’s data interpretation [16], we suggest that since individuals are not surprised by the increased fluency associated with massed presentations of a stimulus, estimates of liking are generally unaffected. That is, we expect individuals to rate the item in the same way they previously did. Conversely, individuals are more surprised by the unexpected second occurrence of a spaced item, and therefore may tend to increase their liking and semantic processing of it. Accordingly, we expect that estimates of liking should generally increase for the second occurrence of repeated spaced items giving rise to the mere exposure effect.

Second, if spaced items receive, overall, more extensive processing than massed items, they should be remembered better than massed in both younger and older adults (the classical spacing effect).

Third, and most important, memory for spaced items should be qualitatively different across younger and older adults, especially for older adults who show a processing priority for positive information [2, 18–22]. In particular, we expect older adults to remember a higher number of spaced items for which they increased their levels of liking most. Differently, we expect younger adults to show a smaller advantage for spaced items with a positive connotation or, generally, show memory performance that is less dependent on preference.

## Experiment 1: nonwords stimuli

Experiment 1 investigated whether repetition modulates positivity effects, typically found in older adults, by repeating nonwords at study. In particular, younger and older adults rated the liking of a series of nonwords on a 9-point scale (from 1-absolutely not to 9-definitely yes) and subsequently completed a surprise yes–no recognition task. Nonwords at study were presented once or repeated. When repeated, nonwords were repeated either consecutively (massed presentation) or after 6 intervening words (spaced presentation). We also added a control condition of items presented only once. First, we expected participants to evaluate nonwords that were repeated spaced apart more positively in terms of liking compared to nonwords that were repeated in a massed fashion or presented only once. Second, we expected older adults to remember nonwords they liked better than nonwords that they liked less. This pattern should occur for spaced items only. A different pattern of results should be detected in the younger group, who should show greater benefits for repeated spaced items independently of their preference.

### Method

#### Participants

Twenty-seven younger (14 female, ages 18–25, *M* = 21.93) and 27 older adults (15 female, ages 65–80, *M* = 72.19) participated in the study. Participants’ demographic information are shown in Table 1. The younger adults were undergraduates who took part in the study for course credit. Older adults were community-dwelling residents from central Italy. Older adults did not receive monetary reimbursement for their participation. Participants signed an informed consent before enrolling in the study. IRB approval was obtained by the University of Chieti ethical committee. Treatment for memory disorders, brain injury resulting in more than 24-h hospitalization and/or medical issues that could possibly impair cognitive performance (e.g., Alzheimer's disease, multiple sclerosis, and Parkinson's disease) were the exclusion criteria.

**Table 1:** Demographic information for participants in Experiment 1

Measure	Younger adults	Older adults	*p*
	Mean (*SD*)	Mean (*SD*)	
Education (years)	14.2 (2.06)	14.1 (2.51)	0.85
Forward Digit Span	8.44 (2.11)	7.77 (1.42)	0.18
Backward Digit Span	7.88 (1.74)	6.59 (1.15)	< 0.01
Panas POS	31.04 (6.07)	32.95 (4.39)	0.19
Panas NEG	22.25 (3.71)	22.41 (5.73)	0.91
VF	11.19 (2.74)	9.99 (2.51)	0.10
GDS	N/A	9.41 (2.91)	N/A
MMSE	N/A	27.95 (1.03)	N/A

Forward and backward digit span from Mondini et al. [23]; Positive and negative PANAS [24]; VF, Verbal Fluency [23]; GDS, Geriatric Depression Scale [26]; MMSE, Mini Mental State Exam [25]

In addition, all older respondents reported being in excellent mental and physical health and without significant issues with hearing or vision.

A series of cognitive and affective assessment tests were given to volunteers before taking part in the study. We used the classical Mondini et al. [23] forward and backward digit span forms to evaluate working memory capacity, the phonemic version from Mondini et al. [23] to measure verbal fluency, and the Positive and Negative Affective Scale (PANAS) [24], to evaluate positive and negative feelings. Mini Mental State Examination (MMSE) [25] was administered to older adults, to measure general cognitive functioning. Finally, the Geriatric Depression Scale (GDS) [26] was administered to screen older adults for negative thoughts and depression. Tests were administered to determine the absence of cognitive impairment or mood disorders that could affect the task performance.

#### Design

Age was the independent between-subjects variable (younger vs. older adults), and item presentation (single vs. massive vs. spaced repetition) was the independent within-subjects variable. d′ and C statistics were computed to estimate the discrimination and response bias within each condition. Discrimination estimates computed as zHITs-zFalse Alarms and response bias computed as—((z hits minus + z false alarms)/2), were the dependent variable. Response bias (C) by condition was computed with positive values of C representing conservative response bias and negative values a liberal responding bias. Data were adjusted when the proportion of responses equaled 1 or 0 with the correction factor ± ½N with N representing the total number of possible false alarm responses. We considered the percentage of repeated items for which participants increased their preference as an index of the mere exposure effect. We calculated the percentage of spaced items that participants liked most (spaced items that received an increase ≥ 2 points on the second occurrence of spaced items) and we analyzed how remembering was affected (i.e., whether participants remembered a higher percentage of spaced items liked most). With regards to this last comparison, a power analysis revealed that in order for a large effect to be detected (92% chance) as significant at the 5% level, a sample of at least 24 participants would be necessary for one-way between-subjects ANOVA.

#### Materials

We generated 52 nonwords by replacing the consonants of 52 neutral words (mean valence = 5.15) from the Italian Adaptation of the ANEW [27, 28]. These nonwords were rated by an independent group of individuals for pronounceability and ease with which they brought to mind an Italian word. The selected nonwords did not easily bring to mind Italian words but were easy to pronounce. Forty-eight nonwords were divided into four main sets (A, B, C, D), each composed of 12 nonwords. Items were randomly assigned to each set to generate different lists. Each study list contained three sets of items (i.e., A, B and C): Items from Set A were presented once, items from Set B were repeated twice in a massed way (consecutively), whereas those from Set C were repeated after six intervening nonwords (spaced). The set of items not presented during study (i.e., D) was used as distractor items at test. The structure of each study list was obtained by using a template where the 68 single, massed and spaced items were randomly intermixed. Twelve targets were presented once, twelve twice consecutively, and twelve twice but spaced. Four fillers were inserted at the beginning and four at end of the template to avoid primacy and recency effects. At test, all 48 nonwords from the four sets (i.e., A, B, C, and D) were presented in a random order.

#### Procedure

Each item was presented in the center of a computer screen for 3 s. with a 1 s. inter-stimulus interval. The order of nonword presentation at study was randomly intermixed. Learning occurred incidentally. Participants were instructed to silently read each item and rate it on a 9-point scale (from 1-absolutely not to 9-definitely yes) in terms of liking. Participants gave their responses aloud and the experimenter wrote them down. Before beginning the experimental task, participants completed a practice trial to familiarize with the task and were told that some nonwords could be repeated and that they should always follow the same instructions. At test, participants were asked to perform a yes–no recognition-memory test. Studied and new nonwords were presented randomly. Each item remained on the screen until participants responded. They answered by pressing either the key marked *yes,* if they remembered having seen the item during the incidental study phase, or the key marked *no,* if they could not remember having seen the item during the incidental learning phase. The yes–no recognition test immediately followed the study phase. The experimental session lasted about 20 min.

### Results

Results are presented in Table 2.

**Table 2:** d′ as a function of age and type of presentation, mean proportions of the mere exposure effect (MEE) and mean proportions of remembered most liked spaced items in Experiment 1 (standard deviations are in parentheses)

	Type of presentation		MEE	Remembering
Young	Single	.08 (.43)		
	Massed	.46 (.45)	.02 (.50)	
	Spaced	1.04 (.52)	.46 (.12)	.48 (.18)
	FA	.27 (.09)		
Old	Single	− .02 (.47)		
	Massed	.17 (.45)	.07 (.06)	
	Spaced	.61 (.50)	.54 (.07)	.62 (.29)
	FA	35 (.12)		

#### The spacing effect

##### *d*′

A 2 (age group: younger vs. older) × 3 (type of presentation: single, massed vs. spaced) mixed ANOVA on d′ revealed a significant effect of group, *F*(1,52) = 9.79 *p* < 0.01 η_*p*_^2^ = 0.16 as older adults remembered a lower number of items (0.19) compared to younger adults (0.53).

There was a significant effect of type of presentation, *F*(2,104) = 105.67 *p* < 0.001 η_*p*_^2^ = 0.67 In fact, single faces were remembered less than massed and spaced items (single − 0.06, massed 0.31, spaced 0.83). In addition, massed items were remembered less than spaced items (*p*s < 0.001).

Finally, the two-way interaction was not significant *F*(2,104) = 0.89 *p* = 0.41 η_*p*_^2^ = 0.02. We observed a similar pattern of performance across the two groups.

##### Response bias C

A 2 (age group: younger vs. older) × 3 (type of presentation: single vs. massed vs. spaced) mixed ANOVA on response bias computed as—((z hits minus + z false alarms)/2) revealed a no significant effect of group, *F*(1,52) = 0.84 *p* = 0.36 η_*p*_^2^ = 0.01.

There was a significant effect of type of presentation, *F*(2,104) = 105.67 *p* < 0.001 η_*p*_^2^ = 0.67, because response bias for single items (M = 0.55) was more conservative compared to memory for both massed (M = 0.36, *p* < 0.001) and spaced items (M = 0.10, *p* < 0.001). Furthermore, response bias for massive items was more conservative compared to spaced items (*p* < 0.001). The two-way interaction was not significant, *F*(2,104) = 0.89 *p* = 0.41η_*p*_^2^ = 0.02.

#### The mere exposure effect

A 2 (age group: younger vs. older) × 2 (type of repetition: massed vs. spaced) mixed ANOVA on the proportion of items that received an increase in liking at study showed a significant effect of group, *F*(1,52) = 16.5 *p* < 0.001 η_*p*_^2^ = 0.24 as older adults increased their liking for repeated items to a greater extent (0.31) than younger adults (0.24).

There was a significant effect of type of repetition *F*(1,52) = 891.69 *p* < 0.001 η_*p*_^2^: 0.94 because liking for spaced items (0.50) increased more than liking for massed items (0.04).

The two-way interaction was not significant, *F*(1,52) = 1.22 *p* = 0.28 η_*p*_^2^: 0.02. Both younger and older adults showed a similar pattern of performance.

#### The effect of liked spaced items on subsequent memories

A between-subjects one-way ANOVA on the proportion of remembered spaced items that participants liked most over the total of remembered spaced items showed a significant main effect of group, *F*(1,52) = 4.58 *p* < 0.05 η_*p*_^2^ 0.08. In fact, older adults remembered a higher number of spaced nonwords (0.62) that they liked most compared to younger adults (0.47).

## Experiment 2: unfamiliar faces as stimuli

In this experiment, we aimed to clarify whether results reported in Experiment 1 for verbal stimuli could be extended to pictorial stimuli as well such as unfamiliar faces. In fact, whether repetition may increase the liking of unfamiliar spaced faces may have important implication in everyday life. For instance, previous studies showed that repeated angry faces were rated as less negative than novel angry faces [29, 30], highlighting the role of the mere exposure effect in approach-oriented behavior. However, as far as we know, there are no studies with older adults. As in Experiment 1, younger and older adults rated a series of unfamiliar faces in terms of liking and subsequently completed a yes–no recognition memory task. If repetition and liking interact to generate a positive evaluation for faces as well, we expect a positivity bias in memory to occur with spaced faces that participants liked most.

### Method

#### Participants

Twenty-seven younger adults (13 female, ages 18–25, *M* = 23.63) and 27 older adults (14 female, ages 65–80, *M* = 74.15) participated in the study. None of them had participated in Experiment 1, but some of the older adults took part in other experimental sessions in our lab. Participants’ demographic information is summarized in Table 3. The younger adults were undergraduates at the University of Chieti who participated for course credit. Older adults were community-dwelling residents from central Italy. Older adults did not receive monetary reimbursement for their participation. Participants signed an informed consent before enrolling in the study. IRB approval was obtained by the University of Chieti ethical committee. Exclusion criteria included treatment for memory problems or any medical conditions that could affect cognitive functioning. All older participants were in good mental and physical health. The screening was the same as in Experiment 1.

**Table 3:** Demographic information for participants in Experiment 2

Measure	Young adults	Older adults	*p*
	Mean (*SD*)	Mean (*SD*)	
Education (years)	14.70 (2.31)	14.29 (2.03)	0.49
Forward digit span	8.88 (1.42)	8.33 (1.44)	0.16
Backward digit span	7.88 (1.36)	6.48 (1.12)	< 0.01
Panas POS	33.07 (4.32)	34.77 (3.94)	0.13
Panas NEG	23.41 (6.57)	23.62 (6.02)	0.89
VF	11.01 (2.74)	10.03 (2.53)	0.18
GDS	N/A	9.33 (3.05)	N/A
MMSE	N/A	27.95 (0.99)	N/A

Forward and backward digit span from Mondini et al. [23]; Positive and negative PANAS [24]; VF, Verbal Fluency [23]; GDS, Geriatric Depression Scale [26]; MMSE, Mini Mental State Exam [25]

#### Design

As for Experiment 1, we first focused on the spacing effect (single, massed vs. spaced) between the two groups (young vs. old). Then, we compared younger and older adults on the mere exposure effect (increases in liking for massed and spaced items). Finally, we computed the number of spaced items that received an increase in liking and analyzed memory for this type of item across groups.

#### Materials

We selected 52 unfamiliar neutral faces (26 females and 26 males) from the KDEF database [31]. The allocation of items to the study lists was the same as for Experiment 1. Forty-eight faces were divided into four main sets (A, B, C, D), each composed of 12 faces. Items were randomly assigned to each set to generate different lists. The typical study list contained three sets of items (i.e., A, B and C): Items from Set A were presented once, items from Set B were repeated twice in a massed way (consecutively), whereas those from Set C were repeated after six intervening faces (spaced). The set of items not presented during study (i.e., D) was used to provide the distractor items in the test list. The structure of each study list was obtained by using a template where single, massed and spaced faces were randomly intermixed. This template consisted of 68 item presentations. Twelve targets were presented once, twelve twice consecutively and 12 targets were presented twice but spaced apart. Four fillers were inserted at the beginning and four at end of the template to reduce primacy and recency effects. Finally, the test list contained all 48 faces from the four sets (i.e., A, B, C, and D) in random order.

#### Procedure

The experimental timeline and procedure were the same as in Experiment 1. Each face was presented visually in the center of a computer screen for 3 s. with a 1 s. inter-stimulus interval. The order of study presentation was randomly intermixed. Learning occurred incidentally. Participants were told that a face would appear on the screen and were asked to rate each face on a 9-point scale in terms of liking. Participants gave responses aloud and the experimenter wrote them down. At test, participants performed a yes–no recognition-memory test. Studied and new faces were presented randomly. Each item remained on the screen until participants responded. Participants pressed the key marked *yes,* if they remembered having seen the face during the incidental study phase, or the key marked *no,* if they could not remember having seen the face during the incidental learning phase. The recognition memory test immediately followed the study phase and the experimental session lasted about 20 min.

### Results

Results are presented in Table 4.

**Table 4:** d′ as a function of age and type of presentation, mean proportions of the mere exposure effect (MEE) and mean proportions of remembered most liked spaced items in Experiment 2 (standard deviations are in parentheses)

	Type of presentation		MEE	Remembering
Young	Single	.64 (.55)		
	Massed	1.24 (.38)	.02 (.05)	
	Spaced	1.94 (.46)	.54 (.11)	.43 (.12)
	FA	.19 (.09)		
Old	Single	.03 (.45)		
	Massed	.66 (.41)	.04 (.06)	
	Spaced	1.10 (.51)	.57 (.11)	.64 (.16)
	FA	.35 (.13)		

#### The spacing effect

##### *d*′

A 2 (age group: younger vs. older) × 3 (type of presentation: single, massed vs. spaced) mixed ANOVA on d′ revealed a significant effect of group, *F*(1,52) = 44.43 *p* < 0.001 η_*p*_^2^ = 0.46 as older adults remembered a lower number of faces (0.60) compared to younger adults (1.28).

There was a significant effect of type of presentation, *F*(2,104) = 166.85 *p* < 0.001 η_*p*_^2^ = 0.76 In fact, single faces were remembered less than massed and spaced faces (single − 0.34, massed 0.95, spaced 1.52). In addition, massed faces were remembered less than spaced faces (*p*s < 0.001).

Finally, the two-way interaction was not significant *F*(2,104) = 2.43 *p* = 0.09 η_*p*_^2^ = 0.04. We observed a similar pattern of performance across the two groups.

##### Response bias C

A 2 (age group: younger vs. older) × 3 (type of presentation: single vs. massed vs. spaced) mixed ANOVA on response bias computed as—((z hits minus + z false alarms)/2) revealed a no significant effect of group, *F*(1,52) = 1.37 *p* = 0.25 η_*p*_^2^ = 0.03.

There was a significant effect of type of presentation, *F*(2,104) = 166.85 *p* < 0.001 η_*p*_^2^ = 0.76, because response bias for single faces (M = 0.63) was more conservative compared to memory for both massed (M = 0.32, *p* < 0.001) and spaced faces (M = 0.03, *p* < 0.001). Furthermore, response bias for massive faces was more conservative compared to spaced faces (*p* < 0.001). The two-way interaction was not significant, *F*(2,104) = 2.43 *p* = 0.09, η_*p*_^2^ = 0.04.

#### *The mere exposure effec*t

A 2 (age group: younger vs. older) × 2 (type of repetition: massed vs. spaced) mixed ANOVA on the proportion of items that increased in liking at study showed no significant effect of group, *F*(1,52) = 1.85 *p* = 0.18 η_*p*_^2^ = 0.03. The increase in liking was similar across the two groups (younger adults 0.28; older adults 0.30).

There was a significant main effect of type of repetition *F*(1,52) = 749.08 *p* < 0.001 η_*p*_^2^: 0.93 because spaced items (0.55) received a larger increase in liking compared to massed items (0.03).

Finally, there was no significant two-way interaction, *F*(1,52) = 0.06 *p* = 0.88 η_*p*_^2^:0.001 as all participants liked spaced items more.

#### The effect of liked spaced items on subsequent memories

A between-subjects one-way ANOVA on the proportion of remembered spaced items that participants liked most over the total number of remembered spaced items showed a significant main effect of group, *F*(1,52) = 29.7 *p* < 0.001 η_*p*_^2^: 0.36. In fact, older adults remembered spaced faces that received an increase in liking (0.64) more than younger adults (0.43).

## Discussion

This study assessed repetition as a potential source of a positivity bias in memory of older adults. In line with previous studies that aimed to clarify the nature of positivity effects in memory of older adults [32–34], our study suggests that repetition and liking may act together to generate subsequent better memory for items that participants liked most. In particular, we hypothesized better memory for information that received a more positive connotation during encoding especially in older adults. Differently, we did not expect any such priority for liked information in memories of younger adults.

### Repetition effects in memory

We found a main effect of type of repetition in both Experiment 1 and 2. Furthermore, the classical spacing effect was detected in both experiments. When stimuli were repeated, memory performance increased in both younger and older adults. In addition, memory was better when stimuli were repeated spaced apart compared with massed presentations. The major explanation advanced to explain these results relies on repetition priming mechanisms that operate during encoding for both younger and older adults. Repetition priming reduces the processing of the second occurrence of massed items, favoring spaced items that, in this way, receive a total greater amount of processing [5, 6].

When looking at recognition performance in both experiments and across the two groups, we found that recognition memory, in general, increased in Experiment 2 compared to recognition data from Experiment 1 indicating that nonwords were more difficult to remember than faces. This pattern of results mirrors previous data on spacing effects in cued-memory tasks [5, 6] which showed lower performance for unfamiliar verbal items compared to pictorial material. This may be due to the fact that unfamiliar faces may somehow remind participants of semantic details from familiar faces and thus memory may benefit.

Altogether, our findings indicated that spaced repetitions may be used at encoding to sustain memory performance in both younger and older adults. This study also adds to previous studies on spacing effects in aging [35–37] by showing that there are no age-related differences in terms of better memory for spaced items in a single study phase manipulation.

We also found that participants were more liberal on spaced items compared with massed and single items. This finding is in line with previous studies that showed that healthy adults adopt a more conservative response bias when memory judgments become more difficult (as it happens here with single and massed items) [38, 39].

### The mere exposure effects in memory

In both experiments, we replicated mere exposure effects typically found in memory studies [1]. That is, liking for a stimulus increased following repeated exposure to the stimulus.

According to processing fluency theories, this happens because the second time we experience a stimulus, it is processed more easily than novel stimuli. Consequently, repetition increases the ease and speed of processing of the presented stimuli. Such fluency makes stimuli liked better because fluency produces a sense of familiarity and what is familiar is perceived as positive [9]. This is one of the first studies, as far as we know, to show that it is possible to obtain mere exposure effects in a single study phase where items are repeated either consecutively or spaced. In particular, mere exposure effects were obtained only for spaced items, that is, items that were repeated with a number of intervening items in between. This is in line with the mere exposure effect data that evidenced the importance of an interval for the effect to arise. Our data support this hypothesis. In fact, we found that when participants judged target items that were repeated consecutively, the mere exposure effect did not occur. One explanation [16] posits that when items are presented in a massed fashion, the increased fluency associated with consecutive studied items is not surprising and thus it is, generally, not interpreted in terms of increased liking: individuals tend to rate the item in the same way they just did. Conversely, participants do not normally expect to encounter the second occurrence of a spaced item, so when they do, they tend to increase their liking and semantic processing of it.

Another aspect worth noting regards age-related differences. As stated in the Introduction, studies on the mere exposure effect in aging are few [10, 11]. Our study thus adds to literature by showing that it is possible to obtain mere exposure effects in older adults. These data are in line with previous studies on preserved implicit memory mechanisms in aging, e.g., repetition priming [40]. In fact, the mere exposure effect has been considered an example of repetition priming effects [15, 17] that are typically preserved in healthy aging.

A comparison across experiments showed an intriguing pattern of results as older adults tended to show an increase in liking especially with nonwords (Experiment 1), while there were no differences across groups when faces were presented at study (Experiment 2). A tentative hypothesis may be that older adults assigned a greater positive connotation to the second occurrence of a nonword compared to the second occurrence of a face. This may be due to the higher level of fluency and familiarity that a nonfamiliar nonword may convey on its second presentation with respect to faces presentations that may intrinsically be interpreted with a greater level of unfamiliarity than a nonword. However, one may also argue that the opposite should be expected. In fact, we have more experience with perceiving faces in general, so it seems more plausible that the second occurrence of a face would increase feelings of familiarity relative to a nonword. Additional experiments will need to clarify this issue.

### Age-related effects of spacing and mere exposure effects on memory

In line with the hypothesis that repetition and positive affective connotation may interact in memory processes, we found a superiority effect in older adults’ memory for spaced items that were liked most. Differently, younger adults showed a general spacing effect independent of liking. In general, these effects seem to resemble the classical age by valence interaction typically found in positivity effects studies [32, 34, 41] if we assume that what is liked is generally perceived as more positive. In fact, older adults showed a processing priority for items they liked most, whereas younger adults’ memory was less sensitive to increases in liking for spaced items. This pattern of results suggests that older adults’ motivational goals influenced the quality of the affective information to be remembered [2, 22, 42, 43]. In particular, the mere exposure effects for spaced items strengthened older adults’ focus on positive information due to their motivational implications (e.g., positive affect). Our results seem to be in line with those from studies that showed the strength of the positivity effect in memory in older adults [34, 44, 45] by highlighting the role of socio-emotional self-relevant goals in influencing memory performance [2].

Taken together, our results are also noteworthy when the type of studied material is considered. In fact, it has been shown that positivity effects in aging occur more often with meaningful emotionally charged verbal and pictorial material (e.g. words and pictures) [46, 47]. In our case, we found a positivity bias with unfamiliar material (such as nonwords and unfamiliar faces) that was not valenced. Our data seem to point to the role of both perceptually and conceptually driven processes in the generation of positivity bias in aging. Given the incidental learning procedure adopted and the unfamiliar material used, we assumed that more perceptually and implicit mechanisms were at work during encoding (e.g., repetition priming effects) giving rise to older adults’ increases in liking. However, at test, older adults selected only items they liked most, underlining the role of their motivational goals in memory (conceptually driven processes).

### Limitations

Among the limitations of our study, we did not directly test repetition priming effects. A future study should include a session that experimentally primes participants and later investigates memory performance. We are currently running a reaction time experiment in which target nonwords are repeated either in massed or in spaced fashion to explore differences in priming effects between younger and older adults as a function of liking responses.

Another aspect regards the single study phase manipulation of spacing and mere exposure effects. We do not know whether classical spacing and exposure effects paradigms (e.g., distributed in different sessions coupled with long-term retention [48]) may also orient participants’ memory towards the positive pole.

The robustness of effects with different memory tasks should also be investigated. In fact, spacing effects rely on different mechanism according to the type of memory task [49]. For instance, in free recall, one of the major explanations posits that spaced items benefit from more distinctive contextual retrieval cues than massed items due to their greater context variability. In this case, it would be interesting to study whether the mere exposure effect arises and how memory is influenced. Mather and Knight [32] conducted one of the first studies on free recall and repeated testing with a 2-day interval and still found a positivity effect in memory of older adults indicating that repetition may be an important mechanism in the generation of positivity effects.

Finally, it would be also interesting to adopt a remember/know paradigm to better highlight the role of familiarity and recollection in older adults’ greater focus on items they liked most, e.g., whether older adults consciously retrieve contextual details (in this case their liking judgement) that were associated with the item at the time of encoding or just know that the item was on the list even though they cannot recall no specific information about its prior occurrence. Our results seem to point to a recollection-based hypothesis of positivity effects as older adults’ recognition focused on the items, they liked most among a series of items they remembered overall.

## Conclusions

In brief, the primary purpose of the present research was to examine situations that generate positivity effect in aging. We examined whether the type of repetition at encoding has consequences for the generation of positive biases usually exhibited in memory tasks by older adults.

The most significant finding was that when a stimulus was repeated spaced apart and received an increase in liking, older adults’ memory for this type of item increased. Differently, when stimulus presentation (massed or single items) did not lead to an increase in liking, the effect was not observed. More research is needed in order to investigate whether repetition may become more conceptually driven as we age. By investigating perception emotion experiences over the life span, such studies will help further understand age related emotional memory changes. Our results could help researchers to reconsider the role of tacit mechanisms in the production of effects on positivity. The study of repetition and the interaction between memory and emotion is crucial in understanding the mechanisms behind memory deficits in aging [50]. For example, with regards to repetition mechanisms, Alzheimer’s Type Dementia (AD) patients tend to repeat a word, question or activity over and over (the so-called *repetitive questioning* phenomenon) [51, 52]. The reasons may be multifactorial, but cognitive and emotional factors are usually involved. In fact, on one hand, AD patients may feel stressed or anxious about their needs not being met, on the other they are not able to maintain the response in memory. Our study is one of the first to investigate how cognitive and emotional processes may interact via repetition in aging.

## Data Availability

Our dataset from Experiment 1 and 2 are available on OpenScience Framework website project at https://osf.io/bajnv/.

## Abbreviations

GDS: Geriatric Depression Scale
MMSE: Mini Mental State Examination
PANAS: Positive and Negative Affective Scale
